# Early postpartum psychosocial profiles predict parenting maladjustment at 1 year

**DOI:** 10.1038/s41598-026-49031-y

**Published:** 2026-04-27

**Authors:** Asuka Ikeda, Mayumi Nagayasu, Yurina Hoshiko, Goji Nakamoto, Makoto Fujii, Shoko Sugao, Akiko Hanai, Masayo Matsuzaki, Hiroko Watanabe, Masayuki Endo

**Affiliations:** 1 Division of Health Sciences, Graduate School of Medicine, The University of Osaka, Osaka, 565-0871 Japan; 2 Graduate School of Human Sciences, The University of Osaka, Osaka, Japan; 3 Predictive Medicine Special Project (PMSP), RIKEN Center for Integrative Medical Sciences (IMS), RIKEN, Yokohama Kanagawa, Japan; 4 Department of Reproductive Health Nursing, Graduate School of Health Care Sciences, Institute of Science Tokyo, Tokyo, Japan

**Keywords:** Postpartum, Parenting maladjustment, Psychosocial factors, Cognitive–behavioural characteristics, Machine learning, Comprehensive Scale for Parenting Resilience and Adaptation, Diseases, Health care, Medical research, Psychology, Psychology, Risk factors

## Abstract

Parenting difficulties during the first postpartum year are a public health concern. Early screening mainly uses depressive-symptom measures such as the Edinburgh Postnatal Depression Scale (EPDS), which capture concurrent symptoms but may miss broader psychosocial vulnerability linked to later parenting outcomes. We hypothesised that early multidimensional profiling using the Comprehensive Scale for Parenting Resilience and Adaptation (CPRA) would predict later parenting maladjustment. Data from two longitudinal cohorts (*N* = 215) were analysed. Maladjustment was defined as an increase of at least mean + 0.5 standard deviation in the *Psychological Adaptation to Parenting* difficulty score from 1 to 12 months postpartum. Eight predictors, including CPRA domains and demographic factors, were entered into an Elastic Net logistic regression, followed by post-Elastic Net logistic regression. Fifty-repetition Monte Carlo cross-validation identified six stable predictors. The final model showed acceptable, reproducible discrimination (median area under the curve, 0.724; interquartile range, 0.652–0.768). Higher early difficulty in *Mother’s Cognitive and Behavioural Characteristics* was a stable predictor of later maladjustment. Early postpartum CPRA-derived psychosocial profiles may help identify mothers at risk of later parenting maladjustment.

**Supplementary Information:**

The online version contains supplementary material available at 10.1038/s41598-026-49031-y.

## Introduction

Parenting difficulties during the first postpartum year represent an important public health issue^1,2^. Clinical postpartum depression has received substantial attention from the research community; nevertheless, despite not meeting the thresholds for mood disorders, several mothers experience parenting difficulties, including uncertainty, loss of confidence, emotional overload, and challenges in understanding infant cues^3,4^. These difficulties affect maternal well-being, infant development, and family functioning, and are therefore relevant to population health and early prevention strategies^1,5^. In countries such as Japan, where declining birth rates amplify the importance of supporting each family, universal home-visit programs at 1-month postpartum and routine infant check-ups offer unique opportunities for early identification and intervention.

However, current screening practices rely heavily on emotional symptom measures, such as the Edinburgh Postnatal Depression Scale (EPDS)^4,6–8^. Although these tools capture depressive or anxious affect, they do not fully reflect the multidimensional nature of parenting adaptation^1,6,9^. In particular, while the EPDS can capture depressive symptoms at one month postpartum, it is a symptom-focused screening measure and does not directly capture the broader multidimensional nature of parenting adaptation. Depressive symptoms can be associated with some longer-term mother–infant outcomes (e.g. bonding) in longitudinal studies; however, symptom screening alone may be insufficient to characterise broader psychosocial vulnerability relevant to subsequent parenting adjustment^10,11^. Consequently, mothers who appear well-adjusted early on but later deteriorate may remain undetected by symptom-focused screening alone. Furthermore, postpartum maladjustment has been demonstrated by previous studies to be shaped not only by emotional states but also by cognitive–behavioural characteristics, infant temperament, social and family support, and environmental resources^1,4,5,9,12,13^. Nonetheless, existing studies have typically investigated these factors in isolation, limiting their ability to identify complex or subtle patterns of early vulnerability^3,5^. Given that the expression of parental vulnerability is shaped by individual characteristics and the surrounding support environment, early screening must consider personal psychosocial factors and continuity of postpartum support systems. Longitudinal studies with trajectory analysis have consistently reported the existence of a late-onset or postpartum-only psychological distress subgroup^14,15^, reinforcing the need for screening tools that capture underlying psychosocial risk factors.

To address this critical gap and enable a more comprehensive assessment of early parenting vulnerability, our group developed the Comprehensive Scale for Parenting Resilience and Adaptation (CPRA), a multidimensional instrument that consists of 81 items, 21 factors, and five domains (namely, *Child’s Temperament and Health*, *Environmental Resources*, *Perceived Support*, *Mother’s Cognitive and Behavioural Characteristics*, and *Psychological Adaptation to Parenting*)^16^. All CPRA scores are difficulty scores, indicating that higher values consistently denote greater difficulty, burden, or maladaptation^16^. Evidence regarding the multidimensional structure and psychometric performance of CPRA has been reported in development studies and subsequent works, including associations with external measures and the identification of vulnerable subgroups^16,17^. The CPRA enables the assessment of nuanced aspects of early parenting that are not captured by mood-focused instruments, such as cognitive inflexibility, self-regulation tendencies, perceived infant characteristics, and adequacy of support networks^6,9,16–20^.

Although the CPRA has demonstrated utility in cross-sectional assessments, little is known about how early domain-level difficulty scores predict later changes in parental adaptation^16,18^. Additionally, no study has yet employed supervised machine learning to identify reproducible early predictors of postpartum maladjustment using the multidimensional structure of CPRA. As the five CPRA domains represent conceptually related and moderately correlated psychosocial characteristics, traditional regression approaches may be limited by multicollinearity and difficulty in identifying stable early predictors^16^. Elastic Net regression is particularly advantageous for this task as it addresses multicollinearity, performs variable selection, and improves model generalisability, making it suitable for population-based screening models that could be integrated into early postpartum services^21,22^. Supervised machine-learning approaches, particularly penalised regression methods such as Elastic Net, offer advantages for handling correlated predictors, performing variable selection, and improving reproducibility through cross-validation^21,22^. These properties make Elastic Net well-suited for the development of an early screening model using CPRA domain scores^21,22^.

The present study aimed to identify early psychosocial predictors of parenting maladjustment using 1-month CPRA domain scores and a supervised machine-learning approach^16^.

## Results

### Participant characteristics

Participant characteristics are summarised in Table 1. In total, 215 mothers were included in the analysis, among whom 55 (25.6%) and 160 (74.4%) were categorised into the maladjustment and non-maladjustment groups, respectively. The maladjustment group was defined as participants whose increase in the Psychological Adaptation to Parenting score from 1 to 12 months postpartum was greater than or equal to the sample mean plus 0.5 standard deviations. Maternal age did not differ significantly between the two groups (35.4 ± 4.9 vs. 34.7 ± 4.4 years, *p* = 0.201), and the maternal age category was comparable (*p* = 0.196). In contrast, parity showed a significant difference; the maladjustment group had a larger proportion of multiparous mothers than the non-maladjustment group (52.7% vs. 35.0%, *p* = 0.020). The cohort distribution differed significantly, with the LINE cohort constituting a higher proportion of the maladjusted group (70.9% vs. 54.4%, *p* = 0.032).

Baseline (1-month) characteristics of the included and excluded Cohort 2 participants are summarised in Supplementary Table S6. Baseline CPRA domain scores were broadly comparable between these groups, and no material differences were observed across CPRA domains. Baseline characteristics at 1-month postpartum were also compared between the LINE cohort and the hospital cohort to evaluate cross-cohort comparability (Supplementary Table S7). Maternal age was slightly higher in the hospital cohort, and the *Environmental Resources* domain score was also higher, whereas EPDS scores and the remaining CPRA domains were broadly comparable across cohorts.

**Table 1:** Baseline characteristics of participants in non-maladjustment and maladjustment groups (*N* = 215).

Characteristic	Overall (*N* = 215)	Non-maladjustment (*n* = 160)	Maladjustment (*n* = 55)	*p*-value
Maternal age, years	35.1 ± 4.7	35.4 ± 4.9	34.7 ± 4.4	0.201
Maternal age category				0.196
< 35 years	105 (48.8%)	74 (46.3%)	31 (56.4%)	
≥ 35 years	110 (51.2%)	86 (53.8%)	24 (43.6%)	
Parity				0.020
Primiparous	130 (60.5%)	104 (65.0%)	26 (47.3%)	
Multiparous	85 (39.5%)	56 (35.0%)	29 (52.7%)	
Cohort				0.032
LINE cohort	126 (58.6%)	87 (54.4%)	39 (70.9%)	
Hospital cohort	89 (41.4%)	73 (45.6%)	16 (29.1%)	

Note: p-values correspond to overall group comparisons for each categorical variable (chi-square test) or continuous variable (t-test).

### Psychosocial characteristics at 1-month postpartum

The psychosocial characteristics at 1-month postpartum are summarised in Table 2. At 1-month postpartum, the maladjustment group experienced equal or slightly lower psychosocial difficulty than the non-maladjustment group. Regarding depressive symptoms, the maladjustment group had significantly lower EPDS scores (3.93 [2.98–4.88] vs. 6.00 [5.12–6.88], *p* = 0.011).

With respect to the CPRA domains, mothers in the maladjustment group showed modestly lower difficulty in the *Child’s Temperament and Health* domain (*p* = 0.025) and significantly lower difficulty in the *Psychological Adaptation to Parenting* domain (*p* = 0.008). No significant differences in the *Environmental Resources*, *Perceived Support*, and *Mother’s Cognitive and Behavioural Characteristics* domains were noted between the groups.

Taken together, these findings indicate that mothers who later met the maladjustment definition did not necessarily present with greater psychosocial difficulty at 1-month postpartum within the indicators and outcome framework used in the present study. Factor-level comparisons across all 21 CPRA factors are presented in Supplementary Table S2, which shows that the overall pattern was broadly consistent with the domain-level comparisons presented in Table 2.

**Table 2:** Psychosocial characteristics at 1-month postpartum.

Characteristic—1-month postpartum	Non-maladjustment (*n* = 160)	Maladjustment (*n* = 55)	*p*-value
EPDS total score	6.00 [5.12–6.88]	3.93 [2.98–4.88]	0.011
CPRA domains			
*Child’s Temperament and Health*	2.31 [2.22–2.40]	2.12 [2.00–2.24]	0.025
*Environmental Resources*	2.25 [2.17–2.32]	2.23 [2.11–2.35]	0.830
*Perceived Support*	2.24 [2.14–2.34]	2.21 [2.06–2.36]	0.786
*Mother’s Cognitive and Behavioural Characteristics*	2.39 [2.31–2.47]	2.49 [2.36–2.63]	0.183
*Psychological Adaptation to Parenting*	2.35 [2.25–2.45]	2.09 [1.94–2.24]	0.008

Note: Values are presented as mean [95% confidence interval]. p-values were calculated using two-sample t-tests.

### Psychosocial characteristics at 12-month postpartum

The psychosocial characteristics at 12-month postpartum are summarised in Table 3. At 12-month postpartum, a markedly different psychosocial profile emerged. Regarding depressive symptoms, the maladjustment group showed significantly higher EPDS scores (7.04 [5.47–8.60] vs. 4.20 [3.55–4.85], *p* = 0.011).

Across the CPRA domains, mothers in the maladjustment group reported substantially greater psychosocial difficulty in the *Child’s Temperament and Health* (*p* = 0.011), *Perceived Support* (*p* = 0.001), *Mother’s Cognitive and Behavioural Characteristics* (*p* < 0.001), and *Psychological Adaptation to Parenting* (*p* < 0.001) domains. Differences in the *Environmental Resources* domain were not statistically significant.

Taken together, these findings indicate that despite showing relatively few psychosocial difficulties at 1-month postpartum, the maladjustment group experienced broad and pronounced deterioration across multiple domains at 12 months. Factor-level comparisons across all 21 CPRA factors are presented in Supplementary Table S3, which shows that the overall pattern was broadly consistent with the domain-level comparisons presented in Table 3.

**Table 3:** Psychosocial characteristics at 12-month postpartum.

Characteristic—12-month postpartum	Non-maladjustment (*n* = 160)	Maladjustment (*n* = 55)	*p*-value
EPDS total score	4.20 [3.55–4.85]	7.04 [5.47–8.60]	0.011
CPRA domains			
*Child’s Temperament and Health*	2.00 [1.90–2.09]	2.25 [2.07–2.42]	0.011
*Environmental Resources*	2.15 [2.08–2.22]	2.25 [2.13–2.36]	0.156
*Perceived Support*	2.26 [2.16–2.37]	2.55 [2.38–2.72]	0.001
*Mother’s Cognitive and Behavioural Characteristics*	2.37 [2.30–2.45]	2.66 [2.52–2.81]	< 0.001
*Psychological Adaptation to Parenting*	2.04 [1.95–2.13]	2.60 [2.43–2.77]	< 0.001

Note: Values are presented as mean [95% confidence interval]. p-values were calculated using two-sample t-tests.

### Prediction model performance

**Fig. 1:**
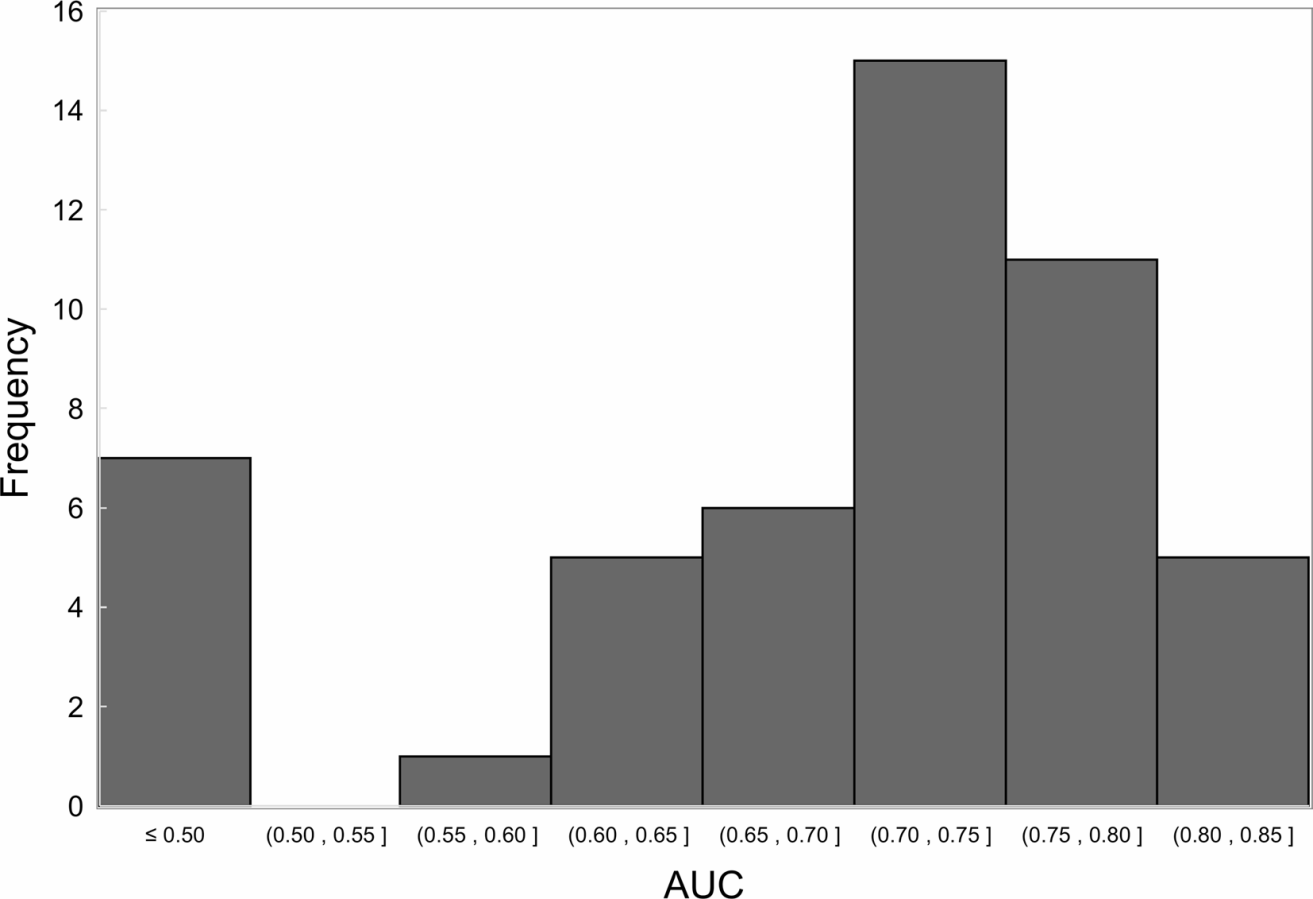
Distribution of test area under the curve (AUC) values for the final 6–predictor post–Elastic Net logistic regression model across 50 Monte Carlo cross-validation runs. This histogram displays the distribution of test AUC values obtained across 50 Monte Carlo cross-validation repetitions. Each bar represents the number of repetitions that produced an AUC within the corresponding interval. The clustering of AUC values around 0.70–0.80 indicates moderate and stable discriminative performance of the post–Elastic Net prediction model. Lower AUC values (≤ 0.50) reflect natural variability due to small test-sample sizes in each split (approximately *n* = 54 per repetition).

The predictive performance of the final six-predictor post–Elastic Net logistic regression model is presented in Fig. 1; Table 4.

Across 50 Monte Carlo cross-validation runs, the median AUC was 0.724 (IQR: 0.652–0.768), indicating an acceptable and reproducible discriminative ability. The stability of the model performance was further confirmed by the narrow IQR of the AUC values.

Coefficients from post–logistic regression models were aggregated across 50 runs to facilitate interpretability and examine the stability and relative contributions of the six retained predictors. All six predictors were selected with a high frequency (40–43/50 runs; Table 4). Greater difficulty in the *Mother’s Cognitive and Behavioural Characteristics* domain showed the strongest positive association with subsequent maladjustment (median β: 1.898, IQR: 1.614–2.137, selection frequency: 43/50). Greater baseline difficulty in the Psychological Adaptation to Parenting domain showed a stable negative coefficient (median β: -1.654, IQR: -1.834 to -1.430, selection frequency: 43/50). Higher difficulty in the *Child’s Temperament and Health* domain exhibited a modest negative association (median β: -0.299, selection frequency: 40/50). Among the maternal characteristics, maternal age ≥ 35 years was negatively associated with maladjustment (median β: -0.353, selection frequency: 42/50), whereas multiparity contributed positively (median β: 0.342, selection frequency: 42/50). The cohort, included as a contextual covariate, was consistently selected (43/50 runs) but was not interpreted as a psychosocial predictor.

**Fig. 2:**
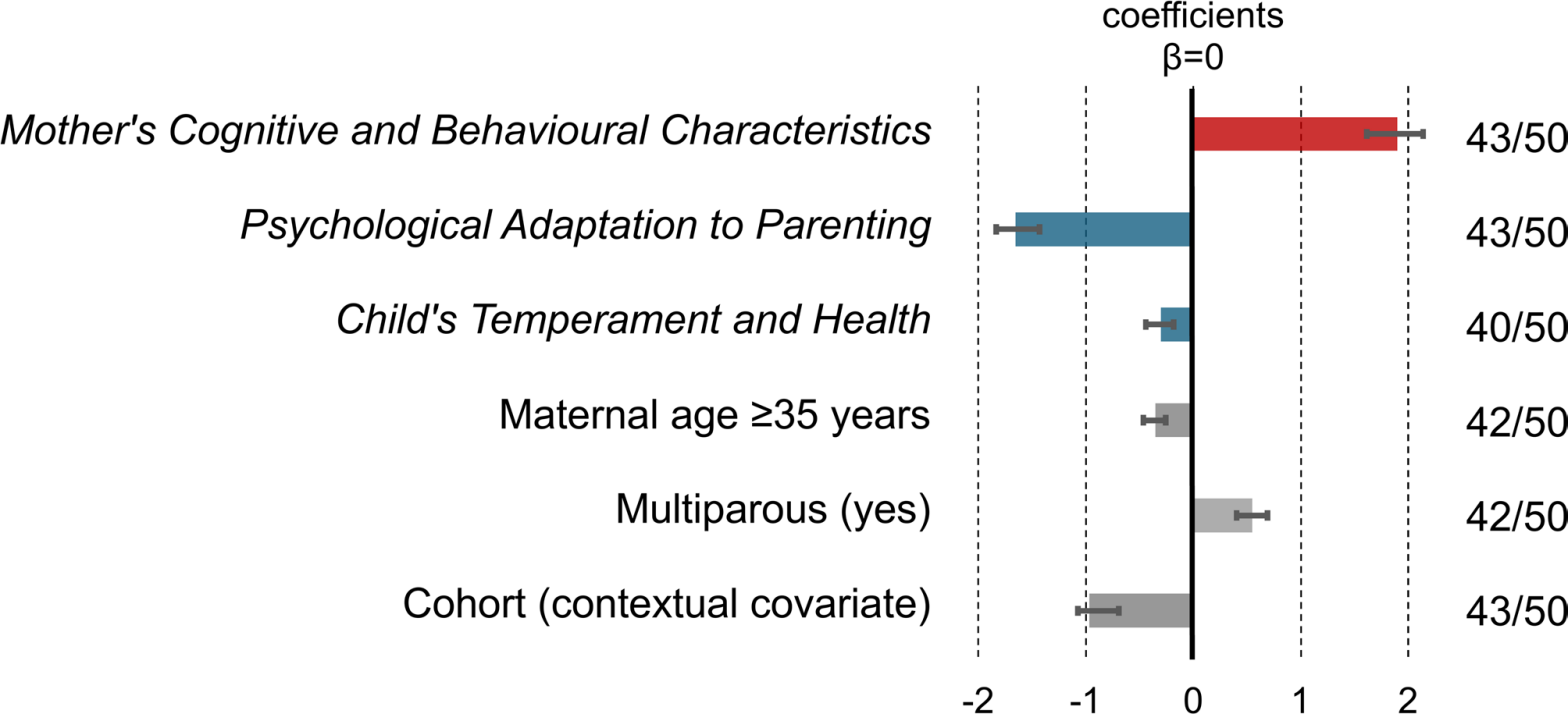
Coefficients and selection stability of the final 6–predictor post–Elastic Net model. This plot displays the median coefficients (β) for six predictors identified through Elastic Net–based predictor selection and estimated using post–Elastic Net logistic regression across 50 Monte Carlo repetitions. Horizontal bars represent interquartile range (25th–75th percentiles), indicating the variability and stability of each estimate. Positive coefficients (to the right of β = 0) correspond to higher likelihood of later maladjustment, whereas negative coefficients indicate lower likelihood. Numbers on the right denote the selection frequency (n/50), reflecting how consistently each predictor was retained across repetitions. CPRA domains are colour-coded according to the direction of association, whereas demographic and contextual covariates are shown in grey and are not interpreted as primary psychosocial predictors.

A visual summary of the model stability and coefficient dispersion across repetitions is provided in Fig. 2, which displays the median coefficients, IQRs, and selection frequencies for the final six-predictor model. As illustrated in the plot, the CPRA-based psychosocial domains related to *Mother’s Cognitive and Behavioural Characteristics* and *Psychological Adaptation to Parenting* were consistently the most influential predictors of later maladjustment.

**Table 4:** Results for the final 6–predictor model identified by Elastic Net and estimated using post–Elastic Net logistic regression (50–run Monte Carlo cross–validation).

A. Coefficients (β) from post–Elastic Net logistic regression			
Predictor: final 6–predictor model	Median β	IQR (25–75%)	Selection frequency (n/50)
*Child’s Temperament and Health*	-0.299	-0.441 to -0.183	40
*Mother’s Cognitive and Behavioural Characteristics*	1.898	1.614 to 2.137	43
*Psychological Adaptation to Parenting*	-1.654	-1.834 to -1.430	43
Maternal age ≥ 35 years	-0.353	-0.463 to -0.256	42
Multiparous (yes)	0.342	0.237 to 0.461	42
Cohort (contextual covariate)	-0.970	-1.075 to -0.693	43

B. Model performance across 50 runs		
Metric	Median	IQR (25–75%)
AUC	0.724	0.652 to 0.768
Sensitivity	0.757	0.572 to 0.833
Specificity	0.640	0.550 to 0.755
Positive predictive value (PPV)	0.444	0.393 to 0.532
Negative predictive value (NPV)	0.913	0.854 to 0.946
Youden’s index–based optimal cut-off	0.236	0.188 to 0.297

Note: Values represent the median and interquartile range (25th–75th percentiles) of coefficients estimated from post–Elastic Net logistic regression models and corresponding performance metrics across 50 Monte Carlo runs. The cohort variable was included as a contextual covariate and was not interpreted as a psychosocial predictor.

### Initial 8-predictor model

To examine whether the exclusion of predictors with low selection frequency improved model reproducibility, we first constructed a full Elastic Net model for predictor selection, including all five CPRA domains, two maternal characteristics, and the contextual covariate (cohort). Across 50 Monte Carlo repetitions, two CPRA domains—*Environmental Resources* and *Perceived Support*—demonstrated low and inconsistent selection frequencies (15/50 and 18/50, respectively). The median AUC of the initial 8-predictor model was 0.695 (IQR, 0.647–0.756).

Supplementary Figure S1 shows the distribution of the AUC values for the initial model. Supplementary Tables S4, S5 summarise the coefficients, selection frequencies, and performance metrics.

### Additional analyses

To reduce potential coupling and regression-to-the-mean effects inherent to change-score outcomes, we modelled the 12-month *Psychological Adaptation to Parenting* domain difficulty score while adjusting for its baseline (1-month) score; *Mother’s Cognitive and Behavioural Characteristics* remained consistently selected (50/50 runs; median β = 0.344, IQR: 0.303–0.370) (Supplementary Table S8).

At the descriptive level, domain correlations were generally positive, including a moderate positive correlation between Mother’s Cognitive and Behavioural Characteristics and Psychological Adaptation to Parenting (*r* = 0.652; Supplementary Table S9).

In the EPDS-inclusive analyses, the EPDS-only model showed modest discrimination (median AUC: 0.627, IQR: 0.584–0.688), and adding EPDS to the final CPRA-based six-predictor model yielded a small increase in discrimination (median AUC: 0.732, IQR: 0.658–0.767) compared with the CPRA-based model alone (median AUC: 0.724, IQR: 0.652–0.768) (Supplementary Table S10).

We also examined a direction-based definition of worsening (Δ = 12-month − 1-month > 0), classifying 85 of 215 mothers (39.5%) as maladjusted. Under this Δ > 0 definition, the final CPRA-based six-predictor model showed modest discrimination (median AUC: 0.672, IQR: 0.616–0.704), with a median sensitivity of 0.750 (IQR: 0.651–0.860) and specificity of 0.590 (IQR: 0.478–0.649) (Supplementary Table S11).

Finally, in a cohort-specific train–test sensitivity analysis, discrimination remained acceptable when training in one cohort and testing in the other (AUCs: 0.710 and 0.722 across directions, respectively; Supplementary Table S12).

## Discussion

This study found that early postpartum CPRA profiles were associated with later parenting maladjustment during the first postpartum year. Difficulty in the *Mother’s Cognitive and Behavioural Characteristics* domain was identified as the strongest and most stable predictor following Elastic Net–based predictor selection and post–Elastic Net logistic regression modelling, whereas the association involving baseline *Psychological Adaptation to Parenting* required cautious interpretation in light of the change-based outcome definition. Maternal age, parity, and cohort were included as demographic and contextual covariates to adjust for background differences and were not interpreted as primary psychosocial predictors. At 1-month postpartum, EPDS scores were lower in the maladjustment group than in the non-maladjustment group, suggesting that depressive symptom severity at that time point did not clearly flag subsequent deterioration in psychological adaptation. The final model achieved an acceptable and reproducible discriminative performance under the primary outcome specification. Importantly, this study applies supervised machine learning for predictor selection, combined with interpretable post–Elastic Net logistic regression, enabling the identification of stable early predictors of postpartum maladjustment.

The interpretation of the primary outcome requires caution because maladjustment was defined using change in *Psychological Adaptation to Parenting* from 1 month to 12 months postpartum. The strong negative association between baseline *Psychological Adaptation to Parenting* scores and the subsequent change (β = −1.654) is consistent with statistical phenomena such as the law of initial values or regression-to-the-mean^23,24^. As maladjustment was defined using a change score, this negative baseline association should also be interpreted in light of mathematical coupling. However, *Mother’s Cognitive and Behavioural Characteristics*—a domain distinct from baseline adaptation—was identified as a robust positive predictor in the model (β = 1.898). Importantly, this finding was robust in a sensitivity analysis modelling the 12-month Psychological Adaptation to Parenting score while adjusting for its baseline (1-month) score, in which *Mother’s Cognitive and Behavioural Characteristics* remained a stable predictor. This finding suggests that, beyond baseline-related statistical effects, additional psychosocial information contributed to the prediction of later maladjustment. Accordingly, while statistical drift may have influenced the magnitude of the observed change, the identified psychosocial profiles retained predictive relevance for long-term maladjustment. This outcome definition was chosen to identify mothers whose adaptation to parenting worsened over time, rather than to capture cross-sectional difficulty at a single postpartum time point. From a clinical and public health perspective, distinguishing mothers who may later deteriorate from those whose early difficulties remain stable or improve may be important for targeting follow-up support.

Because the primary maladjustment definition relied on a sample-dependent SD-based threshold for the change score, we additionally examined a direction-based definition of worsening (Δ = 12-month − 1-month > 0). Under this alternative definition, discrimination was modest, which is plausible given that a direction-based threshold may capture smaller fluctuations and thereby introduce outcome noise. Importantly, the direction of the key coefficients remained broadly consistent with the primary specification, supporting that the identified psychosocial profile was not solely an artefact of the original threshold choice. Taken together, these findings indicate that predictive performance varies depending on how maladjustment is operationalised, while the main predictive patterns identified in early postpartum CPRA profiles are qualitatively stable across these definitions.

Beyond this outcome-definition issue, one of the more stable findings in our model was the positive contribution of *Mother’s Cognitive and Behavioural Characteristics* to later maladjustment. By contrast, the negative coefficient for baseline *Psychological Adaptation to Parenting* should be interpreted cautiously, given the change-based outcome definition and the potential influence of mathematical coupling and regression-to-the-mean. Sensitivity analyses using a baseline-adjusted outcome specification produced broadly consistent findings, supporting that the predictive contribution of cognitive–behavioural characteristics was not solely driven by this statistical artefact. Previous studies have reported that difficulties in emotion regulation and attentional control are relevant to parenting processes^9,12,16^. Similarly, recent comprehensive evidence indicates that challenges in parental emotion regulation may contribute to intermittent inconsistencies or reduced sensitivity in interactions with children^25^.

These findings suggest that cross-sectional difficulty at 1 month postpartum may not fully capture later worsening in parenting adaptation. Some mothers may not appear highly burdened at the early postpartum stage but may later experience increasing psychosocial difficulties as caregiving demands evolve across the first year^26–28^. Early apparent adjustment may therefore not necessarily indicate sustained resilience throughout the postpartum year. Such trajectories have also been reported in longitudinal studies on postpartum mental health^2,29^. Recent work has also described dual-change patterns in self-efficacy and depressive symptoms, where a small subgroup showed initially low distress, followed by later deterioration^29^. In addition, prior research has documented that parental emotion regulation and self-related capacities fluctuate during the early parenting period^30^. Taken together, these observations suggest that psychosocial functioning in early parenthood is dynamic rather than static, and that low levels of difficulty early in the postpartum period do not necessarily indicate sustained resilience over time.

The negative association between the Child’s Temperament and Health and later maladjustment indicates that greater infant-related difficulty reported at 1-month postpartum was associated with a lower likelihood of subsequent maladjustment. Given the modest magnitude of this coefficient, a cautious interpretation is warranted. Rather than indicating a protective effect, this finding suggests that lower levels of early infant-related difficulty do not necessarily predict favourable longer-term adaptation^31^. Previous research has shown that child temperament is related to maternal stress trajectories and interacts with maternal characteristics^19^, providing a contextual background for interpreting this association.

Support-related domains were not retained as stable predictors, despite their theoretical relevance to postpartum mental health^12^. Longitudinal research has shown that levels of perceived social support vary across the early postpartum period^32^. However, in our dataset, *Environmental Resources* and *Perceived Support* showed minimal variability and almost no group-level differences, providing limited predictive signals for regularised modelling. Consistent with prior evidence that the association between social support and maternal mental health differs by postpartum time point^33^, early support measured at 1-month postpartum may capture situational circumstances specific to that period rather than patterns relevant to longer-term trajectories. In contrast, cognitive–behavioural characteristics exhibited greater variability and clearer associations with later maladjustment, which is consistent with evidence that self-regulatory capacities are closely related to parenting processes^13^. Collectively, these findings indicate that support-related factors may be informative at other stages or in different contexts, but their limited variability at 1-month postpartum constrained their predictive utility in this dataset.

Maternal age ≥ 35 years showed a negative association with later maladjustment in the model; however, age was included as a demographic covariate rather than a primary psychosocial predictor. This association should be interpreted cautiously and not as a direct effect of chronological age itself. Prior research has reported that older maternal age is associated with certain psychosocial characteristics and life circumstances that may be relevant to postpartum adaptation^34^. Similarly, parity was included as a demographic covariate to adjust for background parenting experience, rather than being interpreted as a direct psychosocial predictor in the present study. Previous studies have reported associations between multiparity and maternal psychological strain during the postpartum period^35^. Accordingly, the association between parity and the subsequent maladjustment observed here should be interpreted cautiously as a model-adjusted association rather than a causal effect. Finally, the lower baseline EPDS scores observed in the maladjustment group indicated that depressive symptoms alone may not capture all aspects of early vulnerability. Consistent with multidimensional risk models, cognitive–behavioural tendencies may emerge before affective symptoms, suggesting the value of psychosocial assessments beyond mood-based screening tools^36,37^.

An additional descriptive finding was the widening divergence in psychosocial profiles from 1-month to 12-month postpartum. The maladjustment group showed lower or comparable psychosocial difficulty across multiple domains at 1-month but exhibited the highest levels of difficulty, reduced perceived support, and greater depressive symptoms at 12-month. This widening divergence indicates distinct adaptation trajectories, consistent with longitudinal evidence demonstrating late-emerging or increasing postpartum distress patterns^14,15,28^, and highlights the potential value of early multidimensional assessment beyond emotional symptoms alone^36^.

The final post–Elastic Net logistic regression model, following Elastic Net–based predictor selection, achieved an acceptable discriminative performance (AUC: 0.724) with reproducible results across repeated cross-validations. The relatively high negative predictive value (NPV: 0.913) suggests that the model may be useful for identifying mothers who are unlikely to experience later maladjustment. In contrast, the moderate positive predictive value suggests that factors not captured by the early psychosocial profiles included in the present model may be relevant to later maladjustment. These findings indicate that early psychosocial profiles provide one component of information relevant to identifying later maladjustment^38^. This level of discrimination is broadly consistent with the accuracy ranges reported for established postnatal screening tools. For example, a systematic review of the EPDS reported an operational summary receiver operating characteristic (ROC) Q* value of approximately 0.68^37^.

Additional analyses further supported cautious interpretation of the main findings. EPDS-inclusive analyses showed that an EPDS-only model had modest discrimination, and that adding EPDS to the CPRA-based model resulted in only a small improvement in AUC. Accordingly, CPRA-based multidimensional profiling may provide predictive information relevant to subsequent maladjustment alongside symptom screening, rather than replacing it. In addition, a baseline-adjusted 12-month outcome sensitivity analysis yielded consistent selection of *Mother’s Cognitive and Behavioural Characteristics*, supporting the robustness of this predictor under an alternative outcome specification. Finally, cohort-specific train–test validation showed acceptable discrimination when training in one cohort and evaluating performance in the other (AUCs: 0.710 and 0.722 across directions, respectively), supporting reasonable robustness across the two recruitment contexts within this study.

Most previous postpartum screening studies have relied on measures of depressive symptoms such as the EPDS^2,4,8^. While depression screening remains essential, these affective symptom measures may have modest predictive accuracy in practice, with reported operational performance in the moderate range (Q* ≈ 0.68)^37^, and they do not fully capture several determinants of parenting adaptation, including cognitive inflexibility, attentional control, social perceptual processing, and parenting confidence^12,13,25^. Other multidimensional instruments, such as the Parenting Stress Index (PSI), have also been used to assess parenting-related psychosocial stress across multiple domains and have been applied to examine parenting adjustment and parent–child functioning^39,40^. Previous studies using such multidimensional parenting measures have also explored associations with subsequent parenting outcomes and parent–child functioning, suggesting that multidomain psychosocial assessments may provide useful information beyond symptom-based screening alone. The present findings extend previous work by suggesting that multidimensional psychosocial assessment may provide useful information for identifying mothers at risk of later maladjustment, including some mothers who do not appear highly burdened at 1 month postpartum. By applying supervised machine learning to CPRA domain scores, this study enabled the reproducible identification of early predictors while addressing multicollinearity and selection instability. These results highlight the potential value of CPRA-based multidimensional assessments for early postpartum risk stratification.

This study benefited from the integration of two independent cohorts, a comprehensive psychosocial assessment using the full CPRA instrument, and a rigorous machine-learning framework with repeated cross-validation. Baseline characteristics at 1-month postpartum were compared between the two cohorts to assess comparability prior to pooled analyses. Although the cohort distribution differed between groups, cohort membership was included as a contextual covariate to consider the structural differences between recruitment settings. The higher rate of maladjustment observed in the LINE cohort may reflect unmeasured differences between recruitment settings. Unlike the hospital cohort, which maintained a connection with a university hospital, the mobile survey participants represented a broader community sample without routine contact with a specific clinical facility. Consequently, inclusion of the cohort variable allowed partial adjustment for recruitment-related differences. In cohort-specific train–test validation, discrimination remained acceptable when training in one cohort and evaluating performance in the other, supporting the robustness across the two recruitment contexts within this study.

Nonetheless, external validation in single-cohort or population-based samples is important to minimise potential cohort-related biases and clarify generalisability beyond the present recruitment contexts. One additional limitation is the potential for selection and retention bias, as the analytic sample was restricted to mothers who provided complete CPRA responses at both 1-month and 12-month postpartum. Those who completed both assessments may represent a more engaged, stable, or survey-adherent subgroup, whereas mothers with greater burden or less stable circumstances may have been under-represented. Therefore, the generalisability of the findings to the broader postpartum population should be interpreted with caution. Despite the acceptable discriminative ability observed, parenting maladjustment may also be influenced by broader environmental, interpersonal, and developmental factors that were not captured in the present dataset^12,34,41^. Future research could incorporate partner and infant characteristics, longitudinal trajectories of psychosocial change, and fine-grained factor-level CPRA indicators to improve predictive precision and interpretability.

Overall, early postpartum CPRA profiles may provide useful information for identifying mothers at risk of later parenting maladjustment. In particular, early multidimensional assessment may help characterise psychosocial patterns associated with later deterioration, although interpretation should remain cautious with respect to outcome definition and generalisability. These findings support the potential value of CPRA-informed multidimensional assessment for early risk stratification and follow-up planning in routine postpartum care.

## Methods

### Study design

This study integrated data derived from two longitudinal cohorts to identify early predictors of worsening difficulty in the *Psychological Adaptation to Parenting* domain during the first postpartum year. In both cohorts, the CPRA was administered at approximately 1-month and 12-month postpartum.

### Cohort 1: LINE-based longitudinal survey

A web-based longitudinal survey was conducted using LINE, a mobile messaging application that is widely used in Japan. Participants were informed about the survey through brochures and QR codes distributed in collaboration with municipalities and obstetric clinics, and those who wished to participate voluntarily registered through the project’s official LINE account. The Cohort 1 survey started in August 2023, and responses collected up to October 2025 were used for the present analysis. Automated invitations prompted mothers to complete the CPRA at predefined postpartum time points (1 and 12 months). Because cohort 1 is an ongoing open-registration programme, many registered mothers had not yet reached 12 months postpartum at the time of data extraction. Therefore, the analytic sample reflects mothers who met both predefined postpartum windows and pre-specified completeness criteria rather than a conventional retention rate from a fixed inception cohort.

For the 1-month assessment, responses submitted within 0–54 days postpartum were included. When multiple responses were available, the response closest to postpartum day 30 was selected. The distribution of 126 mothers ranged from 4 to 54 days (median: 30 days, interquartile range [IQR]: 30–41 days).

For the 12-month assessment, responses submitted within 280–457 days postpartum were included. When multiple responses were available, the response closest to postpartum day 365 was retained. The distribution of 126 mothers ranged from 280 to 457 days (median: 368 days, IQR: 365–456 days). Overall, 37% (*n* = 47/126) of the mothers responded exactly on postpartum day 365, reflecting the automated reminder system. The analytic sample for Cohort 1 comprised 126 mothers who met both predefined postpartum windows and provided complete CPRA responses at both the 1-month and 12-month assessments.

### Cohort 2: University Hospital Cohort

A university hospital cohort was recruited from University Hospital A between January 2020 and September 2022. Women were enrolled during pregnancy and followed up prospectively. Mothers who consented to participate completed the web-based CPRA assessments at approximately 1-month and 12-month postpartum. In addition to the CPRA, postpartum follow-up at approximately 1 month included other questionnaires (e.g. EPDS). The study protocol designated these two time points as fixed time windows; however, the actual timing of the assessments varied owing to the manual follow-up procedures.

For the 1-month and 12-month assessments, the responses ranged from 28 to 55 days and from 361 to 396 days postpartum, respectively. Of the 192 enrolled mothers, 168 responded to the postpartum follow-up at around 1 month (including questionnaires other than the CPRA). Among them, 118 completed all 81 CPRA items at 1 month. The remaining 50 mothers did not provide complete CPRA data at 1 month, either due to non-response or partial completion. Of the 118 mothers with complete CPRA data at 1 month, 89 also completed all 81 CPRA items at 12 months and were included in the final analysis, whereas 29 did not provide complete CPRA data at 12 months. The baseline characteristics of the included and excluded participants are summarised in Supplementary Table S6.

To evaluate comparability between the two recruitment contexts prior to pooled analyses, baseline characteristics at 1-month postpartum were additionally compared between the LINE cohort and the hospital cohort (Supplementary Table S7).

### Inclusion and exclusion criteria

This study included participants who completed the CPRA at 1-month and 12-month postpartum and provided complete responses to all 81 CPRA items at each time point. However, those with missing CPRA items, implausible birthdates, inconsistent postpartum timing, and pregnancy-period responses (for the LINE cohort) were excluded.

### Ethical considerations

This study was conducted in accordance with the Declaration of Helsinki and was approved by the Institutional Review Board of Osaka University Hospital (approval number 19290; 23 August 2023). Informed consent was obtained from all participants.

### Measures: CPRA

The CPRA is an 81-item instrument rated on a 5-point Likert scale, with all items coded such that higher scores indicate greater difficulty or maladaptation^16^. The items of the CPRA, which assess psychosocial aspects of early parenting, are organised into 21 factors. These factors, in turn, are conceptually grouped into five domains: *Child’s Temperament and Health*, *Environmental Resources*, *Perceived Support*, *Mother’s Cognitive and Behavioural Characteristics*, and *Psychological Adaptation to Parenting*. These domains capture distinct but interrelated dimensions of parenting, including perceived infant difficulty; availability of material, logistical, and familial resources; adequacy of emotional and instrumental support; maternal cognitive–behavioural tendencies (e.g. attentional control, flexibility, emotion regulation); and psychological adaptation encompassing confidence, coping capacity, positive feelings toward the child, and self-esteem. The *Psychological Adaptation to Parenting* domain reflects maternal psychological well-being, which is closely associated with parenting. Psychological adaptation to the parenting role has been conceptualised in maternal–child health research as maternal role attainment or becoming a mother, involving the development of maternal identity, confidence in infant care, and psychological adjustment to parenting responsibilities^42,43^. The structure of the factors and content of the items are detailed in Supplementary Table S1. The development and subsequent applications of CPRA have been reported previously, including evidence supporting its multidimensional structure and associations with external measures^16,17^. These psychometric and structural findings were established in independent developmental samples rather than in the cohorts analysed in the present study. The present study used the established domain structure for prediction modelling and did not aim to re-validate the factor structure within these cohorts.

Domain-level difficulty scores were calculated as the mean of the constituent items (after reverse scoring, when needed) and used as primary predictors, as they summarised coherent psychosocial constructs while reducing dimensionality and multicollinearity. Factor-level scores were used only for descriptive analyses and were not included in the prediction model. This decision prioritised interpretability and parsimony for potential use in routine postpartum services and reduced model complexity, as including all 21 factor-level scores would increase dimensionality and risk of overfitting given the current sample size.

### Outcome: parenting maladjustment

The primary outcome was parenting maladjustment, defined as the worsening of the *Psychological Adaptation to Parenting Difficulty* score from 1-month to 12-month postpartum. Mothers whose change score was at least mean + 0.5 standard deviation (SD) of the full sample were classified as the maladjustment group (*n* = 55/215, 25.6%). This threshold was used as an operational, sample-dependent definition to identify relatively pronounced worsening within the current dataset and was not treated as a validated clinical cut-off. All other mothers were classified into the non-maladjustment group (*n* = 160).

### Prediction model, validation, and optimisation

A 50-repetition Monte Carlo cross-validation was performed to evaluate the model robustness and optimise the selection of predictors^22^. In each repetition, the dataset was randomly split at the participant level into a 75% training set and a 25% test set. No additional class-weighting or resampling was applied during model fitting; the Elastic Net and post-selection logistic regression models were fitted using the observed class distribution in the analytic sample. To avoid degenerate splits due to class imbalance, we required the test set to include at least five positive and five negative cases in each repetition; otherwise, the split was re-sampled. Within each training set, the penalty parameter λ was selected through three-fold internal cross-validation^21^. Eight predictors were included in the model: five CPRA domains as primary psychosocial predictors, maternal age category and parity as demographic covariates, and cohort membership. Maternal age was summarised as a continuous variable in baseline descriptive statistics to describe the sample distribution. For prediction modelling, age was categorised (< 35 vs. ≥ 35 years) to align with a commonly used definition of advanced maternal age and to support interpretability in clinical and public health contexts^44^. Cohort membership was treated as a contextual covariate to adjust for differences in recruitment settings and data collection modalities. All participants were pooled to maximise the sample size, model reproducibility, and stability. The stability of the predictors was evaluated using the selection frequency across 50 repetitions. Six out of eight predictors showed high and consistent selection frequency (40/50) and were thus retained as the final set of stable predictors for subsequent post–Elastic Net logistic regression modelling.

### Final model specification

The final post–Elastic Net logistic regression model included six stable predictors: three CPRA domains (*Child’s Temperament and Health*, *Mother’s Cognitive and Behavioural Characteristics*, and *Psychological Adaptation to Parenting*), maternal age category, parity, and cohort (contextual covariate). Inclusion of the cohort covariate was deemed necessary to ensure model robustness by statistically adjusting for baseline heterogeneity between the two integrated cohorts. Model performance metrics, including the area under the ROC curve (AUC), sensitivity, specificity, and predictive values, were summarised using medians and IQRs across 50 Monte Carlo simulations. All analyses were performed using Stata version 18 (StataCorp, College Station, TX, USA). This study was reported in accordance with the Transparent Reporting of a multivariable prediction model for Individual Prognosis or Diagnosis (TRIPOD) guidelines.

### Additional analyses

To address concerns regarding mathematical coupling and regression-to-the-mean when using change-score outcomes, we performed a sensitivity analysis modelling the 12-month *Psychological Adaptation to Parenting* domain difficulty score while adjusting for its baseline (1-month) domain difficulty score under the same framework.

To provide descriptive context for interpretation of the domain-level findings, we also examined correlations among the five CPRA domains at 1 month postpartum.

As an additional analysis, we evaluated EPDS at 1-month postpartum (total score) under the same 50-repetition Monte Carlo cross-validation framework. Specifically, we fitted (i) an EPDS-only model including the EPDS total score with the same demographic/contextual covariates (maternal age category, parity, and cohort) and (ii) a combined model adding the EPDS total score to the final CPRA-based six-predictor model.

To evaluate robustness to the maladjustment definition and to reduce reliance on a sample-dependent SD-based cut-off, we conducted an alternative outcome-definition analysis using a direction-based criterion, classifying mothers as maladjusted if the *Psychological Adaptation to Parenting* domain difficulty score increased from 1 month to 12 months postpartum (Δ = 12-month − 1-month > 0).

To assess robustness across the two integrated cohorts and address external validity concerns, we conducted a cohort-specific train–test sensitivity analysis (cohort-fixed validation), training the model in Cohort 1 (LINE-based longitudinal survey) and evaluating performance in Cohort 2 (university hospital cohort), and then repeated this in the reverse direction using the primary maladjustment definition (change score ≥ mean + 0.5 SD).

## Supplementary Information

Below is the link to the electronic supplementary material.

Supplementary Material 1

## Data Availability

The data that support the findings of this study are available from the corresponding author upon reasonable request. The data are not publicly available because they contain information that could compromise the privacy of the research participants.
