# Supplementary material for: Early postpartum psychosocial profiles predict parenting maladjustment at 1 year
Source: Sci Rep. 2026 Apr 27;16:21750. doi: 10.1038/s41598-026-49031-y (PMC13358059; doi:10.1038/s41598-026-49031-y)
Supplement: Supplementary file 1 — Supplementary Material 1 [file 41598_2026_49031_MOESM1_ESM.pdf]

Supplementary information

Title

Early postpartum psychosocial profiles predict parenting maladjustment at 1 year: A machine-learning analysis using the Comprehensive Scale for Parenting Resilience and Adaptation (CPRA)

Authors

Asuka Ikeda [(1)]*, Mayumi Nagayasu [(1)], Yurina Hoshiko [(1)], Goji Nakamoto [(1)], Makoto Fujii [(1)], Shoko Sugao [(2)], Akiko Hanai [(3)], Masayo Matsuzaki [(4)], Hiroko Watanabe [(1)] and Masayuki Endo [(1)]

Affiliations

[(1)] Division of Health Sciences, Graduate School of Medicine, The University of Osaka, Osaka, Japan

[(2)] Graduate School of Human Sciences, The University of Osaka, Osaka, Japan

[(3)] Department of Artificial Intelligence Medicine, Graduate School of Medicine, Chiba University, Chiba, Japan

[(4)] Department of Reproductive Health Nursing, Graduate School of Health Care Sciences, Institute of Science Tokyo, Tokyo, Japan

*** Corresponding author:** Asuka Ikeda

Division of Health Sciences, Graduate School of Medicine, The University of Osaka

Osaka 565-0871, Japan

E-mail: ikeda@sahs.med.osaka-u.ac.jp

Telephone: +81-6-6879-2681

Supplementary Table S1. Comprehensive Scale for Parenting Resilience and Adaptation (CPRA): Questionnaire Items, Domains, and Factors Overview

Domain	Factor	Item
Child's Temperament and Health	Child's Temperament and Health	My child often seems uncomfortable when I hold him/her
		My child is generally in a good mood*
		My child cries often
		My child gets sick easily
		My child does not eat much (including feeding and baby food)
Environmental Resources	Relationship with the Medical Staff	I think the healthcare workers are reliable*
		I can trust the healthcare workers*
		I can communicate well with the healthcare workers at the regular health checkup for my child*
		I can't decide on the family doctor for my child
	Partner Temperament	My husband (partner) is a difficult person
		My husband (partner) is very particular about something
		I am often at the mercy of my husband (partner)
		My husband (partner) can control his emotions well*
	Parental Autonomy	My parents are good at time management*
		My parents are good at keeping things tidy and in order*
		My parents can take care of themselves*
		My parents are healthy*
	Partner Autonomy	My husband (partner) is good at time management*
		My husband (partner) is good at keeping things tidy and in order*
		My husband(partner) can take care of himself*
		My husband (partner) is healthy*
	Child-Rearing/Long-Term Care Burden	(If you have an older child) the older child needs much care
		(If you have an older child)The older child is regressing
		(If you have an older child) The older child has an illness or disability
		I care for my parents or grandparents
Perceived Support	Husband's/Partner's Support	My husband (partner) takes care of our child*
		My husband (partner) does housework*
		My husband(partner) is uncooperative in raising children, and I feel lonely
		I feel loved by my husband(partner)*

	Parental Support	My parents help me with childcare*
		I can rely on my parents for childcare and housework*
		My parents often gives me advice about childcare*
		I have someone outside my family who can help me raise my child*
	Lack of Psychological Support from Husband/Partner	My husband (partner) takes care of our child, but I sometimes feel lonely
		My husband (partner) does not understand me as a mother
	Sufficient Social Support	I have a good relationship with my parents (both families)*
		My parents and I have different ways of thinking and methods of raising children, which makes me stressful
		I have a friend who can talk about parenting*
		Relationships with mom friends (including online) are stressful
		I can use the information on the internet about child-rearing*
Mother's Cognitive and Behavioural Characteristics	Inattentiveness	I often forget something
		I often make careless mistakes
		I often miss hearing
		Although I received explanations over and over, I don't understand well
	Emotional Control	I am not bothered or upset when my child doesn't do what I want*
		When I get frustrated, I can calm myself down*
		It is unavoidable that things do not go as planned*
		I often feel so negative that I don't want to see my child's face
	Systemization Urge	I have my ideal form of child-rearing plan, and I want to apply it somehow
		I have an idea that things should be in its right place, and I try to apply it
		I get confused when things don't go as planned
		I want to complete one thing until I am satisfied
	Simultaneous/Overall Processing	I'm good at doing more than one thing at a time*
		I am good at chatting while doing errands*
		With just a little talk, I know what the other person is trying to do*
		It is difficult for me to understand a person's facial expression

	Social Intolerance	I don't understand the explanations of the healthcare workers such as doctors during the regular health checkup for my child
		It is painful for me to wait in a noisy place such as a medical examination waiting room
		I can't calm down unless someone is there
		Even if I receive advice, I want to raise my child in my own way as much as possible
	Attachment Problems	When I was a child, my parents (or major caregivers) didn't take care of me
		When I was a child, I was treated violently by my parents (or major caregivers)
Psychological Adaptation to Parenting	Lack of Self-Confidence	I have a lot of concerns about parenting
		Sometimes I don't know what to do about parenting
		I often get lost when raising children, and I'm worried whether it's correct
		I'm not confident in myself
	Possibility of Coping	I feel that I have time to spend freely*
		I feel that I get relaxed for any length of time*
		I release my stress moderately*
		I think I manage my time well*
	Love for the Child	I love my children*
		I strongly want to protect my children*
		Having children makes me feel warm*
	Self-Esteem	I can overcome difficulties*
		I am able to do what is good for my child's health*
		I can understand my feelings by myself*
		I am proud of myself raising a child*
	Self-Responsibility	I think it's my fault that my child doesn't stop crying
		I feel like my child's cry is blaming me
		I feel empty when I'm raising a child
		I feel like I'm not valued

Note: Responses were collected on a 5-point Likert scale with higher scores indicating increased parenting difficulty. Reverse-scored items are indicated with *

Supplementary Table S2. Comparison of the 21 CPRA factors at 1-month postpartum between the non-maladjustment and maladjustment groups.

Characteristic—1-month postpartum	Non-maladjustment (n = 160)	Maladjustment (n = 55)	p-value
Child's Temperament and Health			
Child's Temperament and Health	2.31 [2.22-2.40]	2.12 [2.00-2.24]	0.025
Environmental Resources			
Relationship with Medical Staff	2.03 [1.92-2.14]	2.03 [1.85-2.21]	0.985
Partner Temperament	2.29 [2.14-2.44]	2.25 [1.99-2.52]	0.814
Parental Autonomy	2.52 [2.40-2.63]	2.51 [2.30-2.72]	0.986
Partner Autonomy	2.90 [2.75-3.05]	2.65 [2.40-2.89]	0.091
Child-Rearing / Long-term Care Burden	1.49 [1.37-1.61]	1.70 [1.50-1.91]	0.070
Perceived Support			
Husband's/Partner's Support	1.81 [1.68-1.95]	1.73 [1.54-1.91]	0.511
Parental Support	2.97 [2.80-3.14]	2.83 [2.53-3.13]	0.404
Lack of Psychological Support from Husband/Partner	2.17 [1.99-2.34]	2.03 [1.77-2.28]	0.403
Sufficient Social Support	2.02 [1.91-2.13]	2.18 [1.99-2.37]	0.158
Mother's Cognitive and Behavioural Characteristics			
Inattentiveness	2.63 [2.49-2.77]	2.84 [2.60-3.07]	0.134
Emotional Control	2.30 [2.18-2.42]	2.29 [2.09-2.49]	0.949
Systemization Urge	2.92 [2.78-3.05]	3.12 [2.88-3.37]	0.132
Simultaneous/Overall Processing	2.49 [2.37-2.62]	2.49 [2.27-2.71]	0.982
Social Intolerance	2.01 [1.91-2.11]	2.12 [1.95-2.29]	0.265
Attachment Problems	1.56 [1.42-1.70]	1.69 [1.44-1.94]	0.265
Psychological Adaptation to Parenting			
Lack of Self-Confidence	2.90 [2.73-3.06]	2.57 [2.34-2.80]	0.041
Possibility of Coping	3.02 [2.89-3.16]	2.70 [2.50-2.91]	0.016
Love for the Child	1.41 [1.29-1.53]	1.32 [1.15-1.49]	0.419
Self-Esteem	2.11 [2.00-2.21]	1.82 [1.65-1.99]	0.008
Self-Responsibility	2.07 [1.94-2.21]	1.82 [1.63-2.02]	0.057

Note: Values are presented as mean [95% confidence interval]. p-values were calculated using two-sample t-tests.

Supplementary Table S3. Comparison of the 21 CPRA factors at 12-months postpartum between the non-maladjustment and maladjustment groups.

Characteristic—12-months postpartum	Non-maladjustment (n = 160)	Maladjustment (n = 55)	p-value
Child's Temperament and Health			
Child's Temperament and Health	2.00 [1.90-2.09]	2.25 [2.07-2.42]	0.011
Environmental Resources			
Relationship with Medical Staff	1.75 [1.64-1.86]	1.79 [1.62-1.96]	0.706
Partner Temperament	2.31 [2.15-2.47]	2.50 [2.22-2.77]	0.247
Parental Autonomy	2.50 [2.38-2.62]	2.54 [2.32-2.75]	0.772
Partner Autonomy	2.73 [2.59-2.87]	2.71 [2.50-2.93]	0.908
Child-Rearing / Long-term Care Burden	1.46 [1.34-1.57]	1.70 [1.50-1.90]	0.037
Perceived Support			
Husband's/Partner's Support	1.92 [1.78-2.06]	2.13 [1.89-2.37]	0.151
Parental Support	3.04 [2.85-3.22]	3.25 [2.96-3.55]	0.226
Lack of Psychological Support from Husband/Partner	2.19 [2.01-2.38]	2.75 [2.43-3.06]	0.004
Sufficient Social Support	1.94 [1.84-2.04]	2.25 [2.05-2.44]	0.004
Mother's Cognitive and Behavioural Characteristics			
Inattentiveness	2.60 [2.45-2.75]	3.02 [2.80-3.24]	0.004
Emotional Control	2.30 [2.18-2.41]	2.59 [2.36-2.82]	0.014
Systemization Urge	2.93 [2.80-3.06]	3.28 [3.07-3.49]	0.009
Simultaneous/Overall Processing	2.53 [2.41-2.66]	2.68 [2.46-2.90]	0.236
Social Intolerance	1.89 [1.79-1.99]	2.22 [2.04-2.40]	0.001
Attachment Problems	1.60 [1.46-1.75]	1.70 [1.47-1.93]	0.500
Psychological Adaptation to Parenting			
Lack of Self-Confidence	2.34 [2.18-2.50]	3.09 [2.82-3.36]	<0.001
Possibility of Coping	2.85 [2.71-2.99]	3.53 [3.32-3.74]	<0.001
Love for the Child	1.16 [1.10-1.23]	1.41 [1.22-1.59]	0.002
Self-Esteem	1.86 [1.76-1.96]	2.15 [1.95-2.35]	0.005
Self-Responsibility	1.79 [1.66-1.91]	2.52 [2.26-2.78]	<0.001

Note: Values are presented as mean [95% confidence interval]. p-values were calculated using two-sample t-tests.

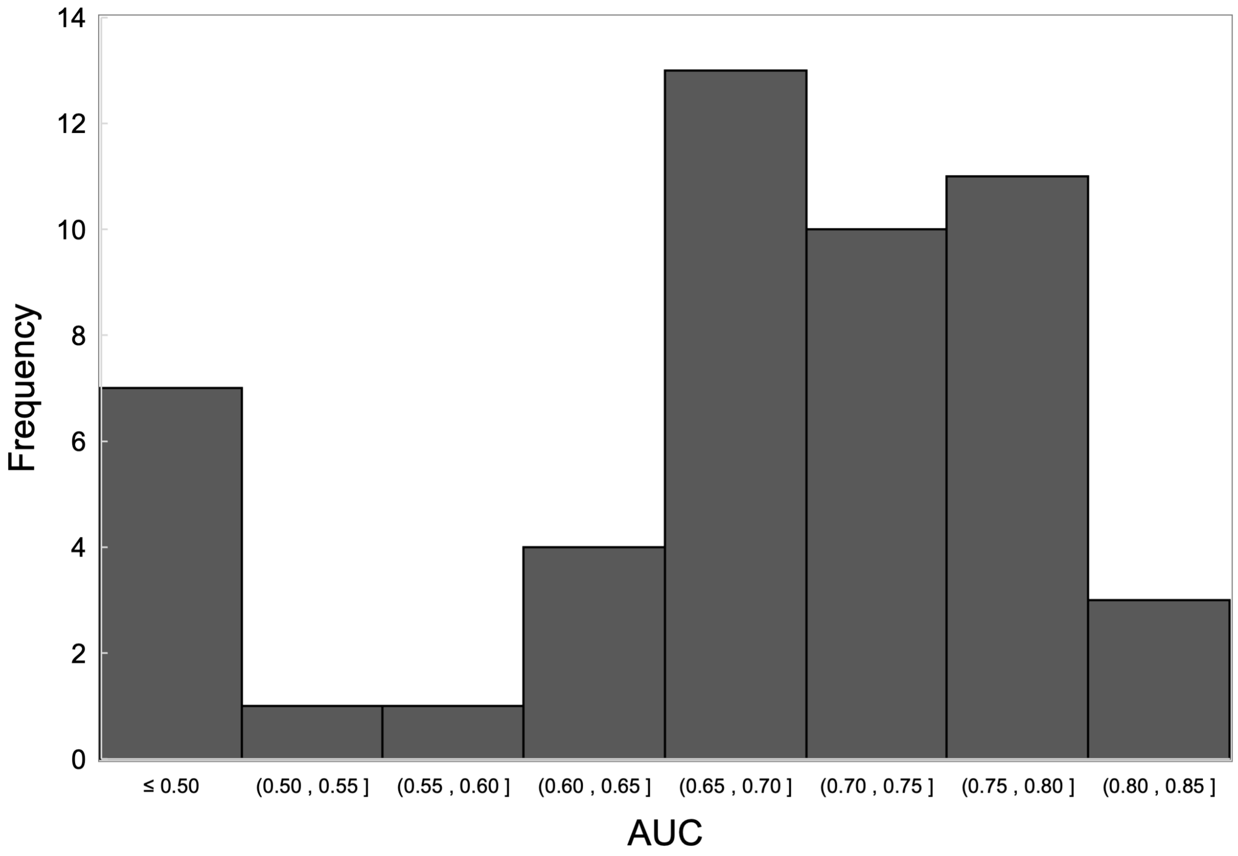

Supplementary Fig. S1. Distribution of test AUC values for the initial 8–predictor post–Elastic Net logistic regression model across 50 Monte Carlo cross-validation runs.

This histogram displays the distribution of test AUC values obtained across 50 Monte Carlo cross-validation repetitions for the initial 8-predictor post–Elastic Net logistic regression model. Each bar represents the number of repetitions that produced an AUC within the corresponding interval. The median AUC (0.695; IQR 0.647–0.756) indicates moderate discriminative performance, with wider dispersion than that observed in the final 6-predictor model. The distribution reflects natural variability in test-sample composition across repetitions (approximately n = 54 per split) and illustrates the reduced stability of the initial model prior to removing predictors with low selection frequency.

Supplementary Table S4. Results for the initial 8–predictor model identified by Elastic Net and estimated using post–Elastic Net logistic regression (50–run Monte Carlo cross–validation).

A. Coefficients (β) from post–Elastic Net logistic regression

**Predictor: initial 8-predictor model**	**Median β**	**IQR (25–75%)**	**Selection frequency (n/50)**
Child's Temperament and Health	-0.320	-0.434 to -0.213	40
Environmental Resources	0.617	0.458 to 0.722	18
Perceived Support	0.267	-0.028 to 0.337	15
Mother's Cognitive and Behavioural Characteristics	1.889	1.620 to 2.088	42
Psychological Adaptation to Parenting	-1.708	-1.915 to -1.533	43
Maternal age ≥35 years	-0.376	-0.543 to -0.256	41
Multiparous (yes)	0.295	0.110 to 0.473	41
Cohort (contextual covariate)	-0.976	-1.122 to -0.800	43

B. Model performance across 50 runs

Metric	Median	IQR (25–75%)
AUC	0.696	0.629 to 0.740
Sensitivity	0.714	0.618 to 0.831
Specificity	0.594	0.526 to 0.711
Positive predictive value (PPV)	0.400	0.360 to 0.462
Negative predictive value (NPV)	0.868	0.815 to 0.929
Youden's index–based optimal cutoff	0.232	0.182 to 0.273

Note: Values represent the median and interquartile ranges (25th–75th percentiles) of coefficients estimated from post–Elastic Net logistic regression models and corresponding performance metrics across 50 Monte Carlo runs. The cohort variable was included as a contextual covariate and was not interpreted as a psychosocial predictor.

Supplementary Table S5. Comparison of predictor stability and model performance between the initial 8-predictor model and the refined 6-predictor model (50 Monte Carlo repetitions)

Model	Predictors included	Selection frequency (n/50)	Median AUC	IQR (25–75%)	Interpretation
**Initial model: 8 predictors**	5 CPRA domains + maternal age ≥35 (demographic covariate) + parity (demographic covariate) + cohort (contextual covariate)	**15–43**	**0.695**	0.647–0.756	Two CPRA domains (*Environmental Resources*, and *Perceived Support*) showed low and inconsistent selection frequency (15–18/50), indicating unstable contributions across repetitions and reduced model reproducibility.
**Final model: 6 predictors**	*Child's Temperament and Health*; *Mother's Cognitive and Behavioural Characteristics*; *Psychological Adaptation to Parenting*; Maternal age ≥35 (demographic covariate); Parity (demographic covariate); Cohort (contextual covariate)	**40–43**	**0.724**	0.652–0.768	Removing unstable predictors increased selection stability and improved overall model reproducibility, yielding a slightly higher and more consistent AUC distribution.

Note: In the initial 8-predictor model, two CPRA domains—Environmental Resources and Perceived Support—showed low and inconsistent stability (selection frequency 15–18/50), whereas the remaining predictors were selected in 40–43 of the 50 repetitions. After removing these unstable predictors, the final 6-predictor model demonstrated a more consistent selection frequency range (40–43/50) and a higher median AUC (0.724 vs. 0.695). These results support the refinement procedure and justify the use of the 6-predictor model as the final, reproducible model.

Supplementary Table S6. Attrition and selection analysis in cohort 2: baseline (1-month) characteristics of included and excluded participants.

Panel A

	**Included (n=89)**	**Excluded A (n=29)**	**p-value**
Maternal age, years	36.0 ± 5.1	34.2 ± 5.0	0.094
Maternal age category			
< 35 years	36 (40.4%)	14 (48.3%)	0.459
≥ 35 years	53 (59.6%)	15 (51.7%)	
Parity			0.335
Primiparous	59 (66.3%)	22 (75.9%)	
Multiparous	30 (33.7%)	7 (24.1%)	
EPDS total score at 1 month, mean ± SD	5.5 ± 5.6	5.2 ± 4.3	0.793
CPRA domains			
Child's Temperament and Health	2.3 ± 0.5	2.4 ± 0.6	0.396
Environmental Resources	2.4 ± 0.4	2.3 ± 0.3	0.054
Perceived Support	2.1 ± 0.6	2.1 ± 0.5	0.504
Mother's Cognitive and Behavioural Characteristics	2.5 ± 0.5	2.5 ± 0.5	0.772
Psychological Adaptation to Parenting	2.3 ± 0.6	2.4 ± 0.6	0.459

Panel B

	**Included (n=89)**	**Excluded B (n=50)**	**p-value**
Maternal age, years	36.0 ± 5.1	35.3 ± 4.0	0.415
Maternal age category			
< 35 years	36 (40.4%)	22 (44.0%)	0.684
≥ 35 years	53 (59.6%)	28 (56.0%)	
Parity			0.229
Primiparous	59 (66.3%)	28 (56.0%)	
Multiparous	30 (33.7%)	22 (44.0%)	
EPDS total score at 1 month, mean ± SD	5.5 ± 5.6	4.3 ± 3.1	0.157

Note: Values are presented as mean ± SD or n (%). All variables were assessed at the 1-month postpartum assessment. Panel A compares included participants (n=89) with those who had complete CPRA data at 1 month but incomplete CPRA data at 12 months (Excluded A, n=29). Panel B compares included participants (n=89) with those who had incomplete CPRA data at 1 month among postpartum follow-up respondents (Excluded B, n=50). P-values were calculated using two-sample t-tests for continuous variables and χ^2 tests for categorical variables (two-sided).

Supplementary Table S7. Baseline (1-month) characteristics of participants by cohort (LINE-based survey vs university hospital cohort).

	Cohort 1 (LINE) (n=126)	Cohort 2 (Hospital) (n=89)	p-value
Maternal age, years	34.4 ± 4.4	36.0 ± 5.1	0.018
Maternal age category			
< 35 years	69 (54.8%)	36 (40.5%)	0.039
≥ 35 years	57 (45.2%)	53 (59.6%)	
Parity			0.142
Primiparous	71 (56.4%)	59 (66.3%)	
Multiparous	55 (43.7%)	30 (33.7%)	
EPDS total score at 1 month, mean ± SD	5.4 ± 5.1	5.5 ± 5.6	0.871
CPRA domains			
Child's Temperament and Health	2.3 ± 0.5	2.3 ± 0.5	0.861
Environmental Resources	2.1 ± 0.5	2.4 ± 0.4	<0.001
Perceived Support	2.3 ± 0.6	2.1 ± 0.6	0.100
Mother's Cognitive and Behavioural Characteristics	2.4 ± 0.5	2.5 ± 0.5	0.065
Psychological Adaptation to Parenting	2.3 ± 0.6	2.3 ± 0.6	0.559

Note: Values are presented as mean ± SD or n (%). All variables were assessed at the 1-month postpartum assessment. Cohort 1 comprises mothers recruited through the LINE-based open-registration programme (n=126), and Cohort 2 comprises mothers enrolled in the university hospital cohort (n=89). P-values were calculated using two-sample t-tests for continuous variables and χ^2 tests for categorical variables (two-sided).

Supplementary Table S8: Baseline-adjusted sensitivity analysis (12-month outcome adjusted for baseline).

Predictor (baseline; 1-month)	Median β (IQR, 25th–75th)	Selection frequency (n/50)
Child's Temperament and Health	-0.052 (-0.089 to 0.031)	15
Mother's Cognitive and Behavioural Characteristics	0.344 (0.303 to 0.370)	50
Psychological Adaptation to Parenting	0.498 (0.460 to 0.532)	50
Maternal age ≥35 years	-0.099 (-0.120 to -0.078)	20
Multiparous (yes)	0.111 (0.090 to 0.138)	43
Cohort (contextual covariate)	-0.107 (-0.133 to -0.076)	34

Note: β indicates the regression coefficient estimated across 50 Monte Carlo cross-validation repetitions and is summarised as the median and interquartile range (25th–75th percentiles). Selection frequency denotes the number of repetitions (out of 50) in which each predictor was selected. The outcome was the 12-month *Psychological Adaptation to Parenting* domain difficulty score, adjusted for its baseline (1-month) score.

Supplementary Table S9. Pearson correlations among CPRA domain difficulty scores at 1-month postpartum.

	Child's Temperament and Health	Environmental Resources	Perceived Support	Mother's Cognitive and Behavioural Characteristics	Psychological Adaptation to Parenting
Child's Temperament and Health	1.000	0.206	0.273	0.302	0.513
Environmental Resources		1.000	0.482	0.474	0.418
Perceived Support			1.000	0.486	0.569
Mother's Cognitive and Behavioural Characteristics				1.000	0.652
Psychological Adaptation to Parenting					1.000

Note: Pearson correlation coefficients (r) are shown. Correlations were calculated using CPRA domain difficulty scores at 1 month postpartum among participants included in the primary analysis (N = 215). Higher scores indicate greater difficulty.

Supplementary Table S10. Model performance with and without EPDS across 50 Monte Carlo cross-validation runs.

Metric	CPRA-only	(i) EPDS-only	(ii) CPRA+EPDS
AUC	0.724 (0.652–0.768)	0.627 (0.584–0.688)	0.732 (0.658–0.767)
Sensitivity	0.757 (0.572–0.833)	0.469 (0.421–0.553)	0.760 (0.615–0.833)
Specificity	0.640 (0.550–0.755)	0.414 (0.381–0.461)	0.598 (0.536–0.770)
PPV	0.444 (0.393–0.532)	0.341 (0.300–0.389)	0.435 (0.371–0.552)
NPV	0.913 (0.854–0.946)	0.842 (0.813–0.877)	0.919 (0.843–0.942)
Youden cut-off	0.236 (0.188–0.297)	0.233 (0.204–0.258)	0.251 (0.196–0.274)

Note: Values are median (25th–75th percentiles) across 50 test sets. Sensitivity, specificity, PPV, and NPV were calculated at the Youden cut-off determined within each test set. Abbreviations: AUC, area under the curve; PPV, positive predictive value; NPV, negative predictive value.

Supplementary Table S11. Sensitivity analysis using an alternative maladjustment definition (Δ > 0): results for the final 6–predictor model identified by Elastic Net and estimated using post–Elastic Net logistic regression (50-run Monte Carlo cross-validation).

A. Coefficients (β) from post–Elastic Net logistic regression

**Predictor: final 6–predictor model (Δ > 0)**	**Median β**	**IQR (25–75%)**	**Selection frequency (n/50)**
Child's Temperament and Health	-0.031	-0.109 to 0.048	18
Mother's Cognitive and Behavioural Characteristics	1.755	1.601 to 1.899	43
Psychological Adaptation to Parenting	-1.729	-1.928 to -1.508	45
Maternal age ≥ 35 years	-0.315	-0.384 to -0.201	32
Multiparous (yes)	0.265	0.131 to 0.370	39
Cohort (contextual covariate)	-0.432	-0.495 to -0.241	25

B. Model performance across 50 runs

Metric	**Median**	**IQR (25–75%)**
AUC	0.672	0.616 to 0.704
Sensitivity	0.750	0.651 to 0.860
Specificity	0.590	0.478 to 0.649
Positive predictive value (PPV)	0.545	0.448 to 0.625
Negative predictive value (NPV)	0.783	0.732 to 0.836
Youden's index–based optimal cut-off	0.372	0.329 to 0.400

Note: Values represent the median and interquartile range (25th–75th percentiles) of coefficients estimated from post–Elastic Net logistic regression models and corresponding performance metrics across 50 Monte Carlo runs. Maladjustment was defined as worsening in the Psychological Adaptation to Parenting domain difficulty score (Δ = 12-month − 1-month > 0). The cohort variable was included as a contextual covariate and was not interpreted as a psychosocial predictor.

Supplementary Table S12. Cohort-specific train–test sensitivity analysis.

Metric	Train Cohort 1 (LINE)→ Test Cohort 2 (Hospital)	Train Cohort 2 (Hospital)→ Test Cohort 1 (LINE)
AUC	0.710	0.722
Sensitivity	0.750	0.897
Specificity	0.548	0.471
PPV	0.267	0.432
NPV	0.909	0.911
Youden cut-off	0.253	0.080

Note: Models were trained in one cohort and evaluated in the other cohort (cohort-fixed train–test validation); this analysis did not use repeated Monte Carlo random splits. Sensitivity, specificity, PPV, and NPV were calculated using the Youden's index–based optimal cut-off determined within each test cohort.
